# Supplementary material for: FastqPuri: high-performance preprocessing of RNA-seq data
Source: BMC Bioinformatics. 2019 May 3;20:226. doi: 10.1186/s12859-019-2799-0 (PMC6500068; doi:10.1186/s12859-019-2799-0)
Supplement: Supplementary file 2 — Archive of FastqPuri. Archive containing all files needed to install and run FastqPuri v1.0.6. Date stamp March 22, 2019. (GZ 47,819 kb) [file 12859_2019_2799_MOESM2_ESM.gz › FastqPuri-1.0.6/html/md_README_trimFilterDS.html]

My Project: trimFilterDS user manual


My Project

trimFilterDS user manual

This program reads two paired end `fastq` files as an input and filters them according to the following criteria:

- Discard/trims reads containing adapter remnants.
- Discards reads matching contaminations (sequences collected in a `fasta` file or in an `idx` file created by `makeTree`, `makeBloom`
- Discards/trims low quality reads.
- Discards/trims reads containing N base callings.

If one of the two rads is discarded, the corresponding paired read is automatically discarded.

## Running the program

Usage `C` executable (in folder `bin`):

Usage: trimFilterDS --ifq <INPUT1.fq>:<INPUT2.fq> --length <READ\_LENGTH>

--output [O\_PREFIX] --gzip [y|n]

--adapter [<AD1.fa>:<AD2.fa>:<mismatches>:<score>]

--method [TREE|BLOOM]

(--idx [<INDEX\_FILE>:<score>:<lmer\_len>] |

--ifa [<INPUT.fa>:<score>:[lmer\_len]])

--trimQ [NO|ALL|ENDS|FRAC|ENDSFRAC|GLOBAL]

--minL [MINL] --minQ [MINQ]

(--percent [percent] | --global [n1:n2])

--trimN [NO|ALL|ENDS|STRIP]

Reads in paired end fq files (gz, bz2, z formats also accepted) and removes:

\* low quality reads,

\* reads containing N base callings,

\* reads representing contaminations, belonging to sequences in INPUT.fa

Outputs 4 [O\_PREFIX]\_fq.gz files per input fastq file containing: "good" reads,

discarded low Q reads discarded reads containing N's, discarded contaminations.

If one read is removed, its corresponding paired read is removed as well.

Options:

-v, --version prints package version.

-h, --help prints help dialog.

-f, --ifq 2 fastq input files [\*fq|\*fq.gz|\*fq.bz2] separated by colons, mandatory option.

-l, --length read length: length of the reads, mandatory option.

-o, --output output prefix (with path), optional (default ./out). Output

-z, --gzip gzips output files: yes or no (default yes).

-A, --adapter adapter input four fields separated by colons:

<AD1.fa>: fasta file containing adapters from read 1,

<AD2.fa>: fasta file containing adapters from read 2,

<mismatches>: maximum mismatch count allowed,

<score>: score threshold for the aligner.

-x, --idx index input file. To be included with any method. 3 fields

3 fields separated by colons:

<INDEX\_FILE>: output of makeTree, makeBloom,

<score>: score threshold to accept a match [0,1],

[lmer\_len]: correspond to the length of the lmers to be

looked for in the reads [1,READ\_LENGTH].

-a, --ifa fasta input file. To be included only with method TREE

(it excludes the option --idx). Otherwise, an

index file has to be precomputed and given as parameter

(see option --idx). 3 fields separated by colons:

<INPUT.fa>: fasta input file [\*fa|\*fa.gz|\*fa.bz2],

<score>: score threshold to accept a match [0,1],

<lmer\_len>: depth of the tree: [1,READ\_LENGTH]. It will

correspond to the length of the lmers to be

looked for in the reads.

-C, --method method used to look for contaminations:

TREE: uses a 4-ary tree. Index file optional,

BLOOM: uses a bloom filter. Index file mandatory.

-Q, --trimQ NO: does nothing to low quality reads (default),

ALL: removes all reads containing at least one low

quality nucleotide.

ENDS: trims the ends of the read if their quality is

below the threshold -q,

FRAC: discards a read if the fraction of bases whose

quality lies below

the threshold -q is over 5 percent or a user

defined percentage in -p.

ENDSFRAC: trims the ends and then discards the read if

there are more low quality nucleotides than the

allowed by the option -p.

GLOBAL: removes n1 cycles on the left and n2 on the

right, specified in -g.

All reads are discarded if they are shorter than MINL.

-m, --minL minimum length allowed for a read before it is discarded

(default 25).

-q, --minQ minimum quality allowed (int), optional (default 27).

-p, --percent percentage of low quality bases to be admitted before

discarding a read (default 5),

-g, --global required option if --trimQ GLOBAL is passed. Two int,

n1:n2, have to be passed specifying the number of cycles

to be globally cut from the left and right, respectively.

-N, --trimN NO: does nothing to reads containing N's,

ALL: removes all reads containing N's,

ENDS: trims ends of reads with N's,

STRIPS: looks for the largest substring with no N's.

All reads are discarded if they are shorter than minL.

NOTE: the parameters -l or –length are meant to identify the length of the reads in the input data. Actually, `trimFilterDS` also copes with data holding reads with different lengths. The length parameter must hold the length of the longest read in the dataset.

## Output description

- `[O_PREFIX1 | O_PREFIX2]_good.fq.gz`: contains reads that passed all filters (maybe trimmed).
- `[O_PREFIX1 | O_PREFIX2]_adap.fq.gz`: contains reads discarded due to the presence of adapters.
- `[O_PREFIX1 | O_PREFIX2]_cont.fq.gz`: contains contamination reads.
- `[O_PREFIX1 | O_PREFIX2]_lowQ.fq.gz`: contains reads discarded due to low quality issues.
- `[O_PREFIX1 | O_PREFIX2]_NNNN.fq.gz`: contains reads discarded due to *N*'s issues.
- `[O_PREFIX1 | O_PREFIX2]_summary.bin`: binary file where information about the filtering process is stored. Structure of the file.
  - filters, `4*sizeof(int) Bytes`: array of int with entries `i = {ADAP(0), CONT(1), LOWQ(2), NNNN(3)}`. A given entry takes the value of the filter it was applied to and 0 otherwise. `filters[ADAPT] = {0,1}`, `filters[CONT] = {NO(0), TREE(1), BLOOM(2)}`, `filters[LOWQ] = {NO(0), ALL(1), ENDS(2), FRAC(3), ENDSFRAC(4), GLOBAL(5)}`, `filters[trimN] = {NO(0), ALL(1), ENDS(2), STRIPS(2)}`.
  - trimmed, `4*sizeof(int) Bytes`: array of integers with entries i = {ADAP(0), CONT(1), LOWQ(2), NNNN(3)}, containing how many reads were trimmed due to the corresponding filter.
  - discarded, `4*sizeof(int) Bytes`: array of integers with entries i = {ADAP(0), CONT(1), LOWQ(2), NNNN(3)}, containing how many reads were discarded due to the corresponding filter.
  - good, `sizeof(int) Bytes`: number of accepted reads (maybe trimmed).
  - nreads, `sizeof(int) Bytes`: total number of reads.

## Filters

#### Adapters

Technical sequences within the reads are detected if the option `--adapters <ADAPTERS1.fa>:<ADAPTERS2.fa>:<mismatches>:<score>` is given. The adapter(s) sequence(s) are read from the fasta files, and then prepended to their respective reads. Then, a 'seed and extend' approach is used to look for overlaps following the same rules followed in the single end case. See `README_trimFilter.md` for details on how two matching subsequences are detected and how the score is computed. The paired reads are correspondingly trimmed, removed or left as is. The following figure describes possible cases:

#### Impurities

Contaminations are removed if a fasta file or an index file are given as an input. The methods provided to look for contaminations work in the very same way as they work for single end data. If one of the reads is discarded, then, the other read is discarded as well. See `README_trimFilter.md` for more details on how the contaminations are handled.

#### LowQ

Again, the detection and trimming/removal of reads containing low quality nucleotides is done following the same procedure as for single end data. We list the options below, see `README_trimFilter.md` for more details.

- `--trimQ NO` or flag absent: nothing is done to the reads with low quality.
- `--trimQ ALL`: all reads containing at least one low quality nucleotide are redirected to `*_lowq.fq.gz`
- `--trimQ ENDS`: look for low quality (below MINQ) base callings at the beginning and at the end of the read. Trim them at both ends until the quality is above the threshold. Keep the read in `*_good.fq.gz` and annotate in the fourth line where the read has been trimmed (starting to count from 0) if the length of the remaining part is larger than `MINL`. Redirect the read to `*_lowq.fq.gz` otherwise.
- `--trim FRAC [--percent p]`: redirect the read to `*_lowq.fq.gz` if there are more than `p%` nucleotides whose quality lies below the threshold. `p=5` per default.
- `--trim ENDSFRAC --percent p`: first trim the ends as in the `ENDS` option. Accept the trimmed read if the number of low quality nucleotides does not exceed `p%` (default `p = 5`). Redirect the read to `*_lowq.fq.gz` otherwise.
- `--trim GLOBAL --global n1:n2`: cut all read globally `n1` nucleotides from the left and `n2` from the right.

**Note:** qualities are evaluated assuming the reads to follow the L - Illumina 1.8+ Phred+33, convention, see Wikipedia. Adjust the values for a different convention.

#### N trimming

We allow for the following options (see README\_trimFilter.md for examples and more details):

- `--trimN NO` (or flag absent): Nothing is done to the reads containing N's.
- `--trimN ALL`: All reads containing at least one N are redirected to `*_NNNN.fq.gz`
- `--trimN ENDS`: N's are trimmed if found at the ends, left "as is" otherwise. If the trimmed read length is smaller than MINL, it is discarded.
- `--trimN STRIP`: Obtain the largest N free subsequence of the read. Accept it if is longer than the half of the original read length, redirect it to `*_NNNN.fq.gz` otherwise.

## Test/examples

The examples in folder `examples/trimFilterDS_SReport/` work in the following way:

1. See folder `fa_fq_files`. The files `EColi_rRNA_DS.read1.fq.gz` and `EColi_rRNA_DS.read2.fq.gz` were created with `create_fq.sh` and contain:
   - 1000 reads of length 50 from `EColi_genome.fa` with NO errors.
   - 5000 reads of length 50 from `rRNA_modified.fa` with NO errrors (rRNA contaminations).
   - Artificially generated reads with low quality score (see `create_fq.sh`)
   - Artificially generated reads with Ns (see `create_fq.sh`).
2. `run_example_TREE.sh`: the code was tested with flags: `../../bin/trimFilter -l 50 –ifq\ –ifq ../fa\_fq\_files/EColi\_rRNA\_DS.read1.fq.gz:../fa\_fq\_files/EColi\_rRNA\_DS.read1.fq.gz –method TREE –ifa ../fa\_fq\_files/rRNA\_modified.fa:0.2:30 \ –trimQ ENDSFRAC –trimN ENDS -o treeDS –adapters \ ../fa\_fq\_files/ad\_read1.fa:../fa\_fq\_files/ad\_read2.fa:2:40 i.e., we check for contaminations from rRNA, trim reads with lowQ at the ends and less than 5% in the remaining part, and strip reads containing N's at the ends.
3. `run_example_BLOOM.sh`: trimFilterDS is run like in 2. but passing a bloom filter to look for contaminations with `score=0.4` and the –trimN STRIP option.
4. With this set up, it is possible to run further customized tests.
5. See the folder `adapters` for examples on adapter contaminations (and its corresponding README file).

NOTE: `rRNA_modified.fa` is the `rRNA_CRUnit.fa` sequence, where we have removed the lines containing N's for testing purposes.

## Contributors

Paula Pérez Rubio

## License

GPL v3 (see LICENSE.txt)


---

Generated by  

 1.8.14
